# ALCAM^+^ stromal cells: role in giant cell tumor of bone progression

**DOI:** 10.1038/s41419-018-0361-z

**Published:** 2018-02-20

**Authors:** Zhenhua Zhou, Yan Li, Xudong Wang, Jingjing Hu, Muyu Kuang, Zhiwei Wang, Song Li, Weidong Xu, Jianru Xiao

**Affiliations:** 1 Department of Orthopaedic Oncology, Changzheng Hospital, Naval Military Medical University (The Second Military Medical University), Shanghai, 200003 China; 2 Cancer Institute, Fudan University Shanghai Cancer Center, Department of Oncology, Shanghai Medical College, Fudan University, Shanghai, 200032 China; 3 Clinical Research Center, Changhai Hospital, Naval Military Medical University (The Second Military Medical University), Shanghai, 200433 China; 4 Department of Thoracic surgery, Fudan University Shanghai Cancer Center, Department of Oncology, Shanghai Medical College, Fudan University, Shanghai, 200032 China; 5 Department of Orthopedics, Changhai Hospital, Naval Military Medical University (The Second Military Medical University), Shanghai, 200433 China

## Abstract

Giant cell tumor of bone(GCTB) is a special benign tumor with variable aggressiveness and recurrence rate. Increasing evidences suggest that a subset of cells called cancer stem cells (CSCs) are present as cancer-initiating cells in a range of malignant tumors. However, the role of CSCs in benign tumor such as GCTB remains unknown, and the connection between the presence of CSCs and biological characteristics of GCTB is unclear. To investigate this issue, we screened a panel of markers of normal stem cells and CSCs and found ALCAM+ stromal cells possessed characteristics of stem-like cells. Subsequently a series of experiments such cell proliferation, migration and invasion assays were performed to investigate the biological characteristics of ALCAM+ stromal cells in vivo and in vitro. The clinical significance of ALCAM expression were further evaluated using Kaplan-Meier analyses. The ALCAM+ GCTB cells showed the stem cell properties of self renewal and had the capacity to differentiate in vitro. The ALCAM+ GCTB cells showed increased resistance for chemotherapy- or radiation-induced cell death. ALCAM knockdown reduced stem/progenitor characteristics in GCTB Cells. Furthermore, ALCAM expression was associated with outcome in GCTB patients. Our work demonstrates for the first time ALCAM+ tumorigenic sub-population within stromal GCTB cells and may represent a potential therapeutic target in aggressive and recurrent GCTBs.

## Introduction

Giant cell tumor of bone (GCTB) is a special primary bone tumor with distinctive biological characteristics, exhibiting three histological different cell types: osteoclast-like multinucleated giant cells, the spindle-shaped, fibroblast-like mesenchymal stromal cell, a round morphology called macrophage-like cells^1^. Although classified as a benign tumor by WHO, GCTB is known for its high local aggressiveness, propensity for local recurrence especially in spine, and infrequent metastases^2^. Furthermore, GCTB is able to evolve into malignant transformation such as sarcomatous changes after irradiation at the primary treatment or spontaneous malignant transformation without radiation therapy^3–5^. Since Cooper first described this tumor in 1818, our understanding of GCTB has progressed, and many attempts have been made to define prognostic parameters for GCTB. However, in spite of available histological system or clinicoradiological system of GCTB used by some pathologists and surgeons, the prognostic significance is still controversially discussed^6–10^. More works should be carried out to further reveal the biological characterization of GCTB and to search for new factors related to GCTB progression that may predict the clinical outcome of GCTB patients.

Cancer stem cells (CSCs) have been defined as a unique subpopulation in tumors that possess the ability to self-renew, develop into any cell in the overall tumor population (multipotency), and proliferate^11–13^. However, most available research reports of CSCs were focus on malignant tumors such as osteosarcoma, hepatocarcinoma and breast carcinoma^14–18^. Do CSCs exist in benign tumors, such as GCTBs? If CSCs exist in GCTBs, from which cell type in GCTB we could identify CSCs? Is the presence of CSCs correlated to biological characteristics of GCTB? In the present study, we chose 20 markers reported to be closely associated with normal stem cells such as mesenchymal stem cell and CSCs to identify markers that were enriched in the potential stem-like fraction of GCTB. We isolated ALCAM^+^ subpopulation from GCTB stromal cells, and performed a series of functional experiments on these cells. We found that ALCAM^+^ stromal cells exhibited the properties of stem-like cells, and ALCAM expression was associated with prognosis of GCTB cases. We hope our findings may provide new insight into the complex mechanisms of GCTBs progression and future clinical applications.

## Results

### Stemness genes expression in GCTB spheres and ALCAM^+^ GCTB cells

**Fig. 1:**
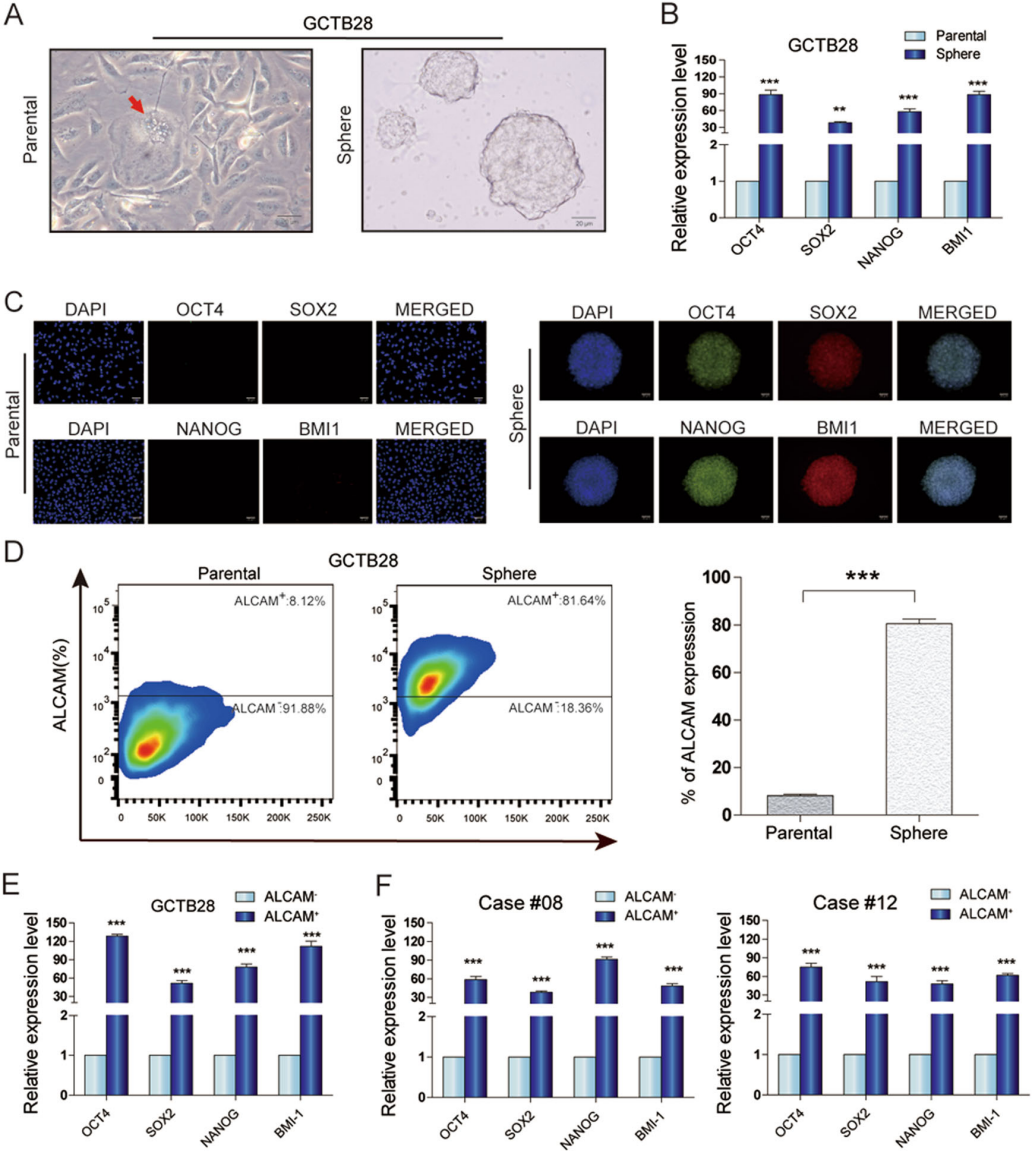
Features of GCTB spheres. **a** Floating spheres derived from GCTB28 cells(left panel) under ultra-low attachment culture conditions. A multinucleated giant cell was indicated by red arrow in the left panel. Spheres of GCTB28 had anchorage dependent growth (right panel). (Scale bar = 20 µm).** b** Comparison of *OCT4*, *SOX2*, *NANOG*, and *BMI1* mRNA expression between GCTB28 parental cells and corresponding spheres cells, respectively. Assessed by using qPCR (t-test, ***p < 0.001; **p < 0.01; *p < 0.05). Error bars represent s.d. from at least three independent experiments. **c** Immunofluorescence staining showed the levels of OCT4, SOX2, NANOG and BMI1 in GCTB28 parental cells (left panel) were lower than that in corresponding sphere cells(right panel). (Scale bar = 20 µm). **d** ALCAM in parental cells and corresponding spheres of GCTB28 cells analyzed by flow cytometry; Independent experiments were repeated three times. Data were presented as mean ± s.d. (***p < 0.001; **p < 0.01; *p < 0.05). **e**, **f** *OCT4*, *SOX2*, *NANOG*, and *BMI1* mRNA in ALCAM^+^ cells from GCTB28 cells (**e**) and clinical samples (cases #08 and #12) (**f**) were greater than that in ALCAM^−^ cells by using qPCR. Independent experiments were repeated three times. Data are means ± s.d. Error bars represent s.d. from at least three independent experiments (***p < 0.001; **p < 0.01; *p < 0.05)

There was sphere formation in GCTB28 cells (Fig. 1a). *OCT4*, *NANOG*, *SOX2*, and *BMI1* gene expression in spheres was significantly higher than in parental GCTB28 cells (Fig. 1b). Immunofluorescence showed that OCT4, NANOG, SOX2 and BMI1 expression was significantly low in parental cells, but high in spheres (Fig. 1c). In addition, 20 candidate cell surface markers in GCTB28 sphere cells and parental cells were expressed and of these, only ALCAM was significantly different between parental cells and spheres (Fig. 1d and Table S1). qRT-PCR data showed that *OCT4*, *NANOG*, *SOX2*, and *BMI1* expression in ALCAM^+^ subsets was significantly higher than in ALCAM subsets in GCTB cells (Fig. 1e, f).

### Chemoresistance and radioresistance of ALCAM^+^ GCTB cells

**Fig. 2:**
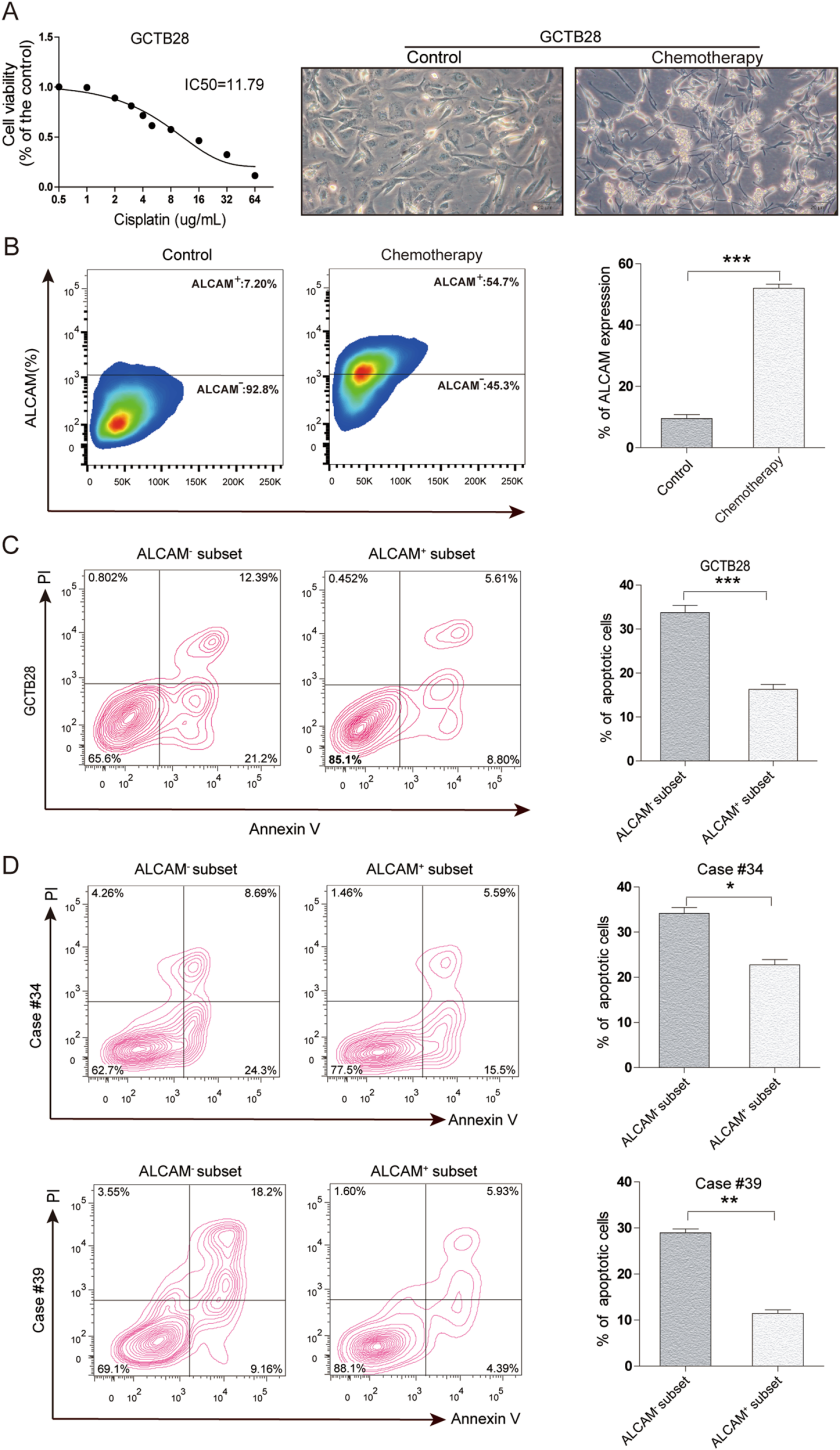
Chemoresistance of ALCAM^+^ GCTB cells. **a** Cell viability of GCTB28 cells after 48 h treatment with cisplatin(DDP) at indicated concentrations by the CCK8 assay(left panel). Light microscopy showed floating dead GCTB28 cells 7 days after chemotherapy with DDP. Surviving cells adhered at the bottom of the culture dish(right panel). (Scale bar = 20 µm). **b** Flow cytometry of ALCAM in GCTB28 cells 7 days after chemotherapy. Independent experiments were repeated three times. Data were presented as mean ± s.d. (***p < 0.001; **p < 0.01; *p < 0.05). **c** and **d**) Flow cytometry for apoptosis in ALCAM^+^ and ALCAM^−^ cells from GCTB28 cells (**c**) and clinical samples (cases #34 and #39) (**d**) after chemotherapy with DDP

After cisplatin(DDP) treatment, most GCTB28 cells underwent apoptosis and floated, but some adherent cells continuted to grow (Fig. 2a). Flow cytometry showed changes in ALCAM expression in surviving GCTB28 cells after chemotherapy. ALCAM expression data appear in Fig. 2b. In addition, we isolated ALCAM^+^ and ALCAM^−^ subsets from GCTB28 cells and measured apoptosis. These data appear in Fig. 2c. We isolated ALCAM^+^ and ALCAM^−^ GCTB cells from case #34 and #39 and apoptosis data for both appear in Fig. 2d. ALCAM expression in GCTB28 cells after 10 Gy radiotherapy data appear in Figure S1A, and compared to before therapy ALCAM expression increased after radiation. Thus, radiotherapy increased the ratio of ALCAM^+^ cells in GCTB28 cells, suggesting that ALCAM^+^ cell subsets might be more tolerant to radiation.

### Biological characteristics of ALCAM + GCTB cells *in vitro*

**Fig. 3:**
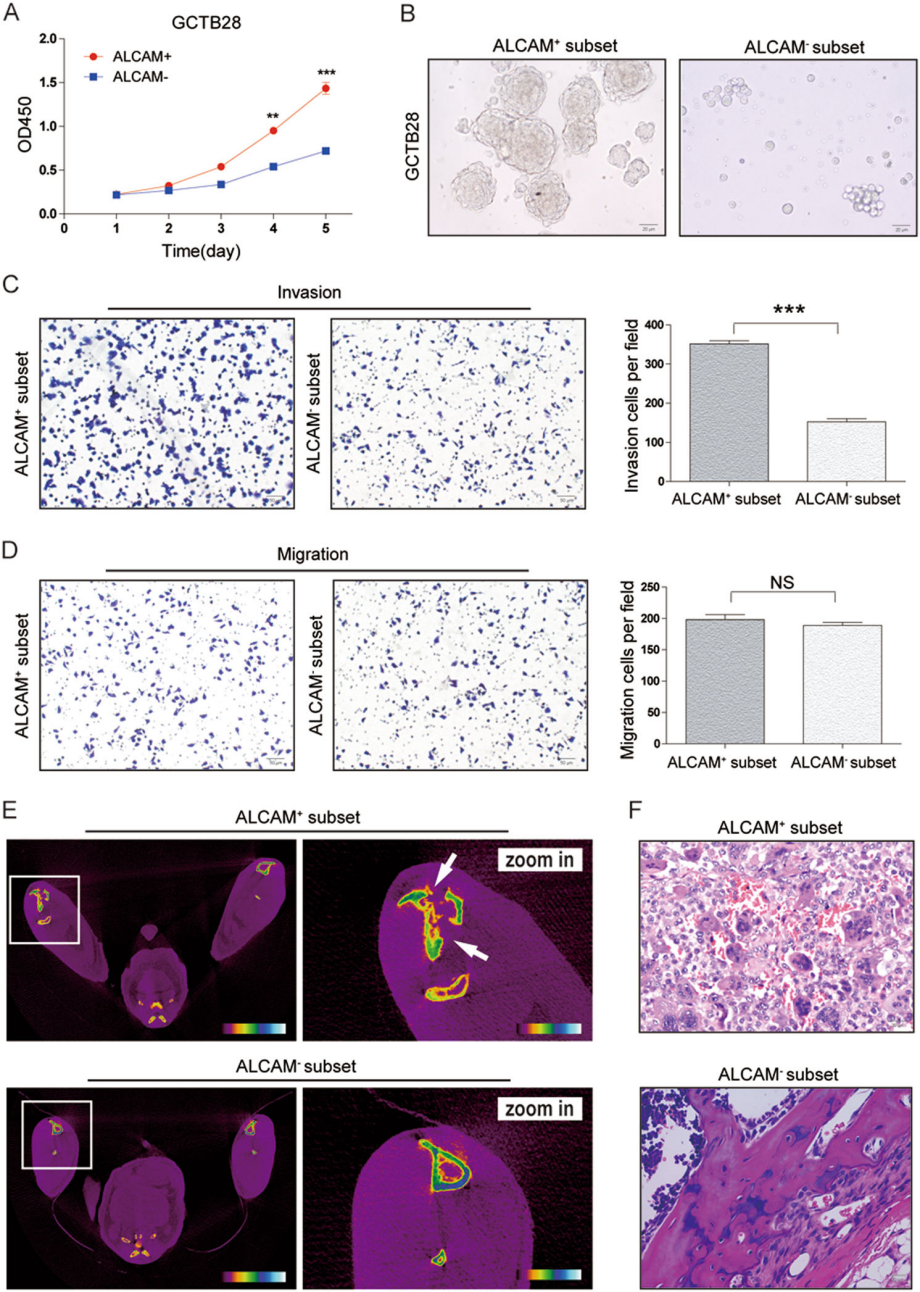
Characteristics of ALCAM^+^ subset iIsolated from clinical GCTB samples. **a** The proliferative rate of ALCAM^+^ cells isolated from GCTB28 cells. Data were presented as mean ± s.d. (***p < 0.001; **p < 0.01; *p < 0.05). **b** ALCAM^−^ cells isolated from GCTB28 cells generated fewer and smaller spheres than ALCAM^+^ cells after 7 days of serum deprivation and ultralow adherent cell culture. (Scale bar = 20 µm). **c**, **d** Compared with ALCAM^−^ cells from GCTB28 cells, ALCAM^+^ cells exhibited stronger (**c**) invasive and (**d**) migratory capacity. Ten high-power fields were selected for comparison. Data were presented as mean ± s.d. (***p < 0.001; **p < 0.01; *p < 0.05). (Scale bar = 50 µm). **e**, **f** ALCAM^+^ cells from GCTB28 cells showed stronger tumorigenic capacity when compared with ALCAM^−^ GCTB28 cells. The ALCAM^+^ cells were isolated from GCTB28 cells and injected subcutaneously into nude mice using the ALCAM^−^cells as a control. ALCAM^+^ and ALCAM^−^ cells with different gradient (500 cells, 1000 cells, 2500 cells and 10000 cells; *n* = 5 in each gradient) isolated from GCTB28 cells were injected subcutaneously into flank of nude mice. See also Table S3. **e** Representative images of ALCAM^+^ and ALCAM^−^ cells-induced tumor formation at 8 weeks after 2.5 × 10^3^ cells injection. The photo shows a tumor nodule at the site of injected ALCAM^+^ cells, but not at the site of injected ALCAM^−^ cells. White arrow indicates severe tibia destruction. **f** Corresponding paraffin-embedded tissue were processed for H&E staining. The histological features of tumor xenograft induced by the ALCAM^+^ cells are comparable to bone tissue by the ALCAM^−^cells (Scale bar = 20 µm). Morphology of mice xenograft tissues showed similar pathological features of human GCTB. Green arrow showed multinucleated giant cells

**Fig. 4:**
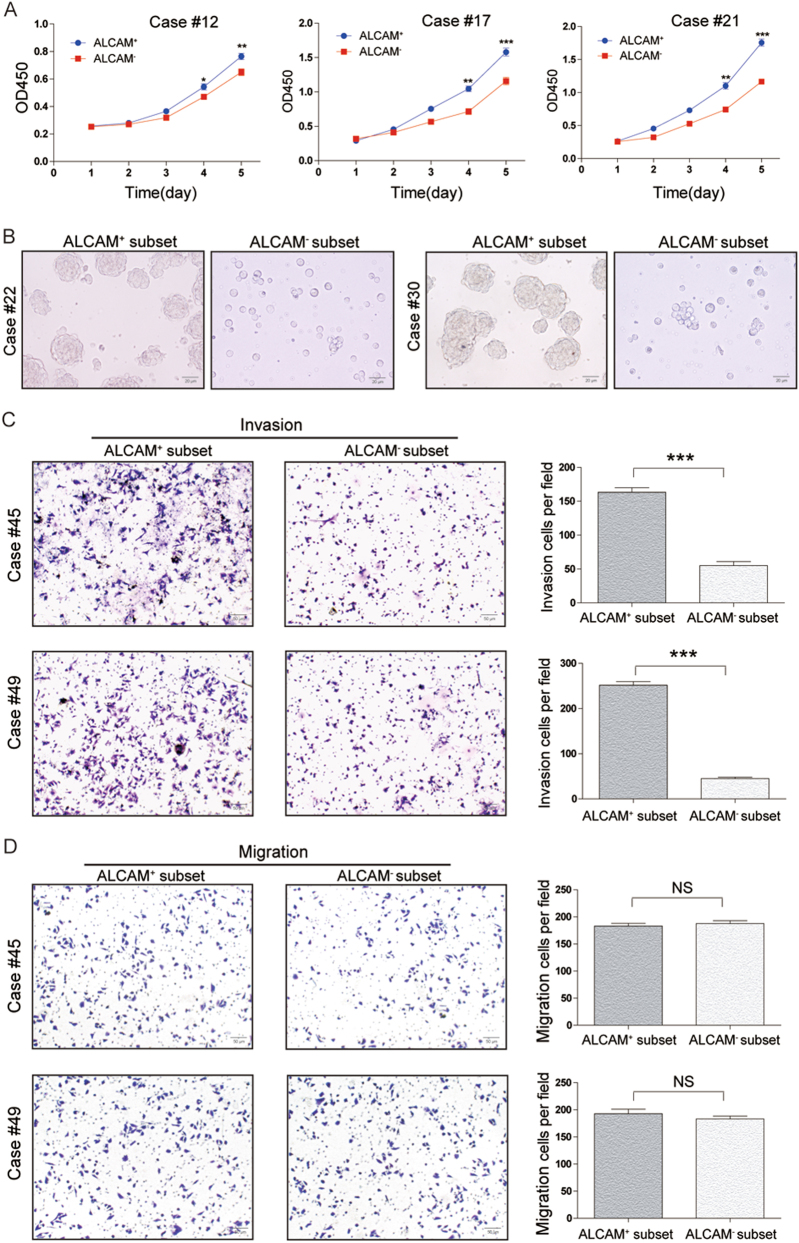
Characteristics of ALCAM^+^ cells isolated from human primary GCTB samples. **a** The proliferative rate of ALCAM^+^ cells isolated from clinical sapmles(cases #12, #17 and #21) was higher than that of ALCAM^−^ cells. Independent experiments were repeated three times. Data were presented as mean ± s.d. (***p < 0.001; **p < 0.01; *p < 0.05). **b** ALCAM^−^ cells isolated from GCTB samples (cases #22 and #30) generated fewer and smaller spheres than ALCAM^+^ cells after 7 days of serum deprivation and ultralow adherent cell culture. (Scale bar = 20 µm). **c**, **d** Compared with ALCAM^-^ cells from GCTB samples(cases #45 and #49), ALCAM^+^ cells exhibited stronger (**c**) invasive and (**d**) migratory capacity. Ten high-power fields were selected for comparison. Data were presented as mean ± s.d. (***p < 0.001; **p < 0.01; *p < 0.05; Scale bar = 50 µm)

Flow cytometry was used for cell-cycle-phase progression analysis in ALCAM^+^ and ALCAM^−^ GCTB28 cells (Figures S2A and S2B). CCK-8 and colony formation assays in GCTB28 cells indicated that the proliferation of ALCAM^+^ cells was higher than that of ALCAM^−^ cells(Fig. 3a, S2C and S2D). For cases #12, #17 and #21, we observed that proliferation of ALCAM^+^ cells was significantly higher than ALCAM^−^ cells (Fig. 4a). In ultra-low adherent cell culture without serum ALCAM^+^ cells isolated from GCTB cells formed more spheres (Figs. 3b and 4b). Invasiveness and migration capacity of subsets of GCTB28 cells and clinical samples (cases #45 and #49) showed that the ALCAM^+^ subset was more invasive (Figs. 3c and 4c). There was no difference in migration capacity of either subset (Figs. 3d and 4d). ALCAM expression in ALCAM^+^ cells decreased in GCTB28 cells in conventional culture conditions (Figure S3A), and sorted ALCAM^−^ cells had stable and low expression of ALCAM two weeks later (Figure S3B), suggesting that ALCAM^+^ cells can differentiate into ALCAM^−^ cells.

### Tumorigenic and metastatic capacity of ALCAM+ subpopulations in GCTB cells

We injected sorted ALCAM^+^ GCTB28 cells with different gradient into the tibial marrow cavity of nude mice and monitored tumor formation in different time point after inoculation. The ALCAM^+^ fraction (2500 cells/injection site) had tumors in mouse tibial marrow cavity after 4–12 weeks, and the ALCAM^−^fraction had no tumors at any time (Fig. 3e, f, Table S3). Injection of 1 × 10^6^ GCTB28 cells containing the ALCAM^+^ subset did not form tumors, and controls had no pulmonary metastases after treatment of GCTB28 cells containing ALCAM^−^ subsets (Figure S4A and S4B).

### ALCAm knockdown reduced stem/progenitor characteristics in GCTB cells

**Fig. 5:**
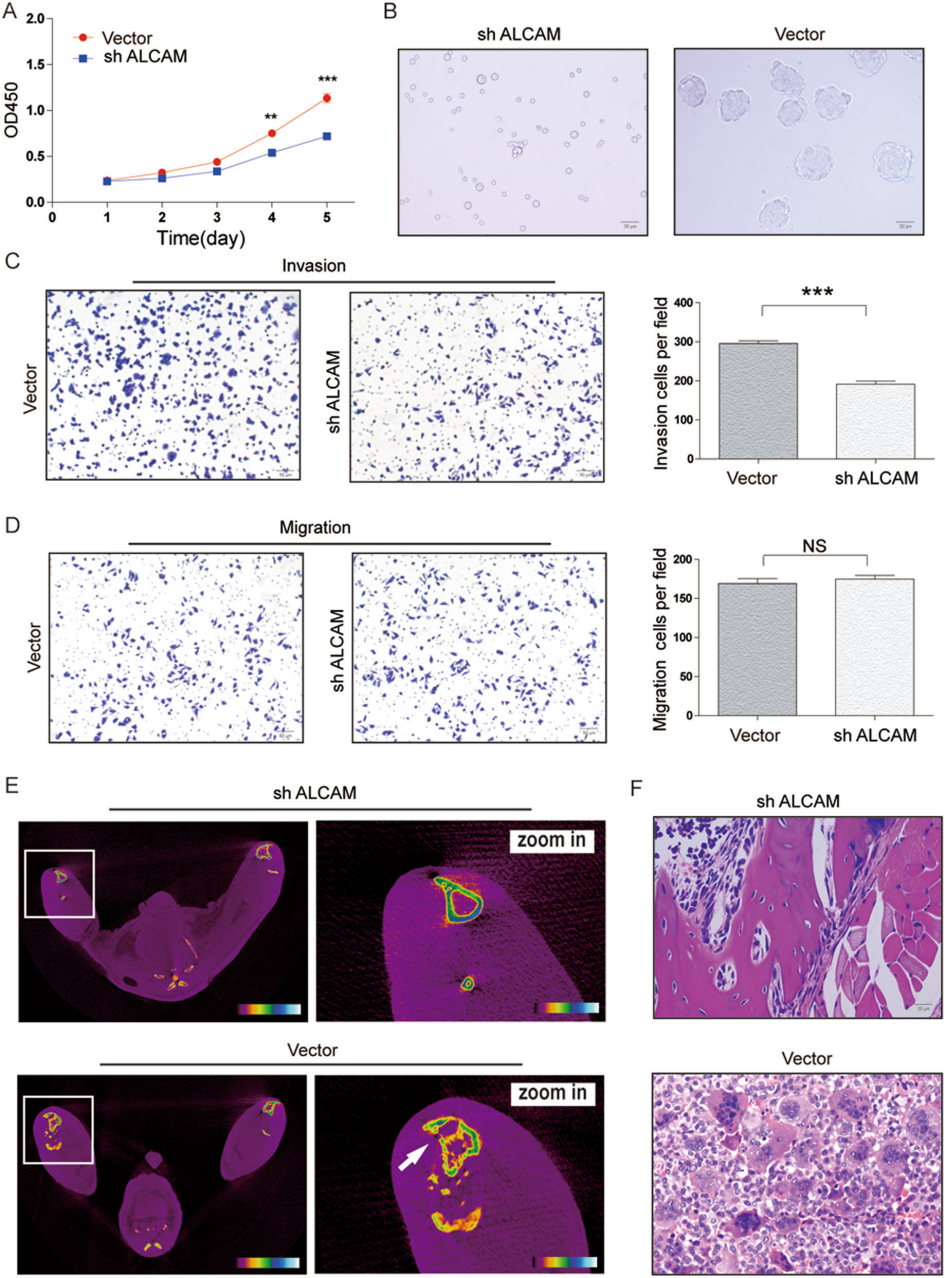
Functional effects of *ALCAM* downregulation in GCTB cells. **a** Knockdown of *ALCAM* inhibited the proliferative rate of GCTB28 cells. Independent experiments were repeated three times. Data were presented as mean ± s.d. (***p < 0.001; **p < 0.01; *p < 0.05). **b** Knockdown of *ALCAM* reduced the size and number of spheres in GCTB28 cells. (Scale bar = 20 µm). **c**, **d** Knockdown of *ALCAM* in GCTB28 cells resulted in significant decrease in (**c**) invasive and (**d**) migratory capacities, respectively. Ten high power fields were selected for comparison. Data were presented as mean ± s.d. (***p < 0.001; **p < 0.01; *p < 0.05). (Scale bar = 50 µm). **e**, **f** *ALCAM* knockdown (sh ALCAM) GCTB cells exhibited reduced tumor-forming incidence when compared with NTC cells. ALCAM knockdown (sh ALCAM) GCTB28 cells and NTC cells with different gradient (1 × 10^3^ cells, 5 × 10^3^ cells, 2.5 × 10^4^ cells and 1 × 10^5^ cells; *n* = 5 in each gradient) were injected subcutaneously into flank of nude mice. See also TableS4. **e** Representative images of sh ALCAM and NTC cells induced tumor formation at 8 weeks after 1 × 10^5^ cells injection. White arrow indicates tibia destruction. **f** H&E staining of paraffin-embedded tumor tissue from mice after orthotopic injection of NTC cells. The histological features of tumor xenograft induced by the NTC cells are comparable to bone tissue by the sh ALCAM cells (Scale bar = 20 µm)

To investigate whether ALCAM play functional roles related to the properties of GCTB stem/progenitor cells, we performed ALCAM knockdown experiments in GCTB28 cells by using lentiviral vectors (Figure S5A). We found that ALCAM knockdown cells proliferated more slowly than the control cells (Fig. 5a). Sphere formation of GCTB28 cells was significantly reduced after knocking out ALCAM, with a significant difference in the sphere number between the ALCAM knockdown and control groups (Fig. 5b). In addition to the reduction in sphere formation, the invasive and cell migration capacities of GCTB28 cells were significantly reduced after knocking out the ALCAM gene (Fig. 5c, d).To assess the tumorigenicity of GCTB cells after knockingdown the ALCAM gene, different numbers of GCTB28 cells were injected into the tibial marrow cavity of mice and underwent imaging observation after the first, second, and third month of inoculation. Injection of 1 × 10^5^ ALCAM knockdown GCTB28 cells showed no tumor formation at different time points (*n* = 0/5 each), whereas injection of 2.5 × 10^4^ GCTB28 cells without ALCAM knockdown induced tumor formation in the second month after inoculation (*n* = 4/5) (Fig. 5e, f, Table S4).

### Clinical significance of ALCAM in GCTB

**Fig. 6:**
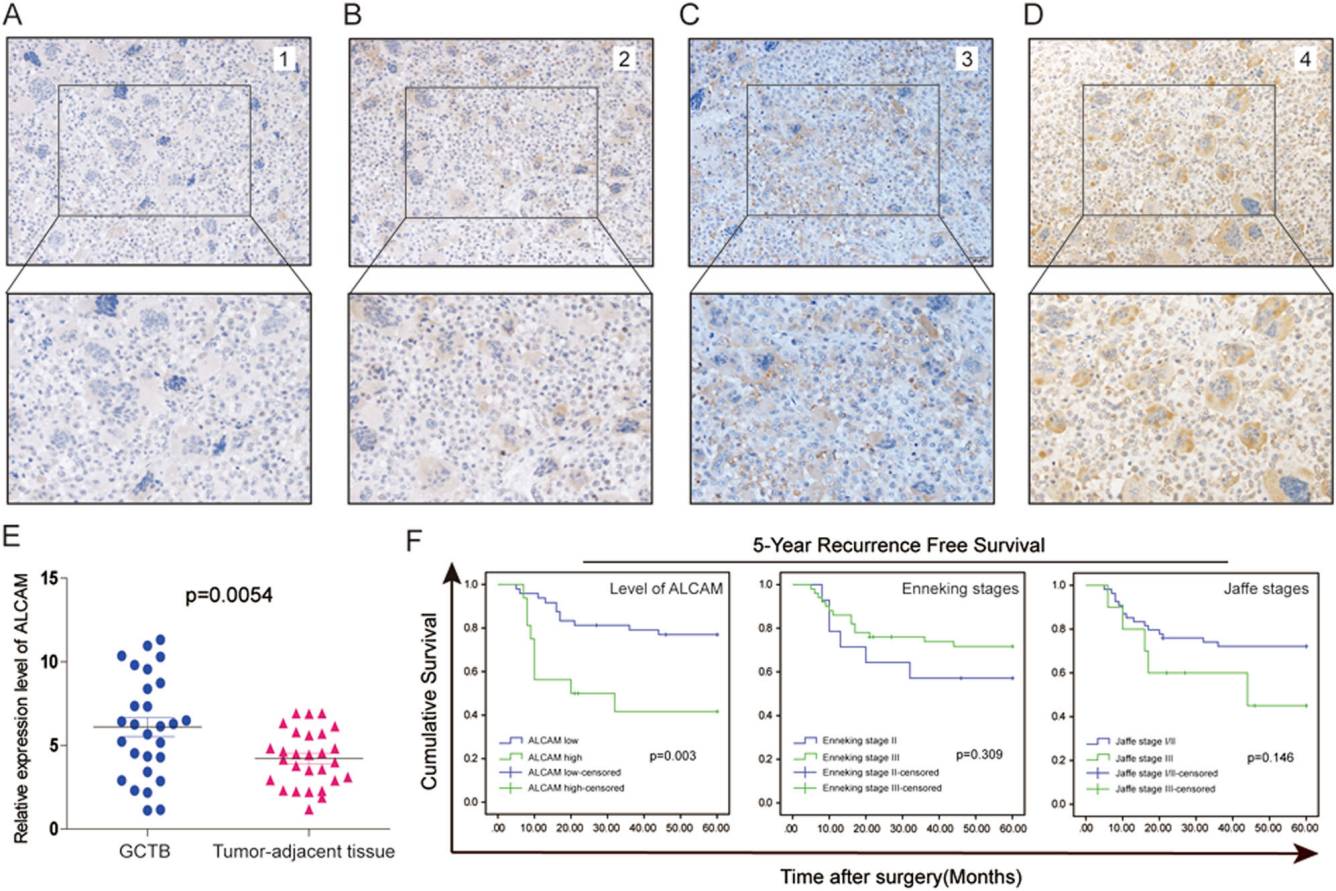
ALCAM expression is correlated with the prognosis of clinical GCTB cases. **a–d** Representative immunohistochemical staining of ALCAM in human GCTB tissues. The cytomembrane staining of ALCAM was categorized and represented as follows: A, score 0; B, score 1; C, score 2; D, score 3. **e** Compared to that in corresponding adjacent tissues, the mRNA expression of *ALCAM* was higher in 28 primary GCTB samples. Independent experiments were repeated three times. Data were presented as mean ± s.d. (*t*-test, ***p < 0.001; **p < 0.01; *p < 0.05). **f** Kaplan–Meier survival curves of overall survival (OS) and disease-free survival (DFS) measured according to the ALCAM expression, Enneking stages and Jaffe stages in clinical GCTB samples

ALCAM expression was upregulated in GCTB compared to nontumorous tissue (Fig. 6e) and immunohistochemical scores (Fig. 6a–d) showed a correlation between ALCAM and the clinicopathologic features (Table S5). There was no correlation between ALCAM in clinical specimens and patients’ age, gender, Enneking stages and Jaffe stages. Tumor relapse, as poor risk factors were associated with ALCAM. Patients with tumors with high ALCAM expression had greater risk of tumor recurrence one year after surgery. Kaplan–Meier data and log rank test results showed that among 64 cases of GCTB, patients with high ALCAM expression had shorter 5-year disease-free survival (DFS) compared to patients. Patients with high ALCAM in tumor tissues had significantly shorter DFS (Fig. 6f).

## Discussion

A number of studies have demonstrated the presence of cancer stem cells in malignant tumors^19–24^, which states that a subpopulation of tumor-initiating cells (TICs) is more tumorigenic than the rest of the tumor cell population and share many characteristics with tissue stem cells, such as self-renewal and differentiation, and may be highly resistant to therapeutics. Eradication of CSCs might represent a promising strategy to explore effective therapies of cancer. Compared malignancies,benign tumors are more likely to be ignored due to being ‘benign’ and not life threatening, which leads to less attention focused on benign tumors and elusive pathogenesis or the underlying molecular process contributing to the development of benign tumors. Accordingly, little is known about what the role of CSCs is in the tumorigenesis and development of benign tumors, and how CSCs in benign tumor are different from their counterparts in malignant tumor. In the present study, we found a novel CSCs-like subset with ALCAM positive and we explored the biological characteristics of this subset in human GCTBs. We hope investigation of the role of ALCAM^+^ subset in GCTB would contribute to better apprehension of this disease development and improved therapy for GCTB.

Our study found that ALCAM^+^ subpopulation sorted from GCTB28 cells proliferated faster than the ALCAM^−^ cells and more fully recapitulated tumor heterogeneity. ALCAM^+^ cells isolated from GCTB tissues also have more powerful oncogenous capacity than ALCAM^−^ cells. Moreover, in immunodeficient mice, ALCAM^+^ cells re-established the GCTB cell hierarchy. It is of note that mice xenograft tumor tissues that generated from stromal cells showed the typical polymorphic morphology of human GCTB. We supposed that most of multinucleated giant cells disappeared in vitro culture for the lack of regulation of the in vivo environment factors which promoted multinucleated giant cells apoptosis or dividing into mononuclear stromal cells. However, when mononuclear stromal cells were injected into immunodeficient mice, stromal cells transformed into or multiple stromal cells fused into multinucleated giant cells. These findings indicated that stromal cells were the predominant force in the onset and development of GCTB.

As we know, most benign tumors lack the ability to invade or metastasize as their malignant counterparts. But GCTB obviously is a very special ‘benign’ tumor, which has a significant tendency to recur locally and rarely may result in the development of lung metastases. Although GCTB is named for the osteoclastlike giant cells, which have been considered the main culprits in bone destruction, the spindle-like stromal cells are believed the neoplastic element of GCTB^1,25^. Previous study suggests GCTB stromal cells originate from mesenchymal stem cells (MSCs) in the bone marrow and exhibit early osteoblastic differentiation^1,25–28^. Our data provided the evidence that cancer stem-like cells population were present within the stromal fraction of GCTB. GCTB stromal cells exhibit cancer stem-like cells features and ALCAM is expressed on the cell surface of a putative cancer stem-like cells sub-population of stromal cells of GCTB. As a matter of fact, although we found that the ALCAM^+^ fraction could generate tumors in the tibial marrow cavity, we failed to obtain a xenograft by subcutaneous or abdominal transfer of ALCAM^+^ GCTB cells in immunodeficient mice(data not shown). And we also didn’t found lung metastasis in immunodeficient mice. These results indicate that tumorigenicity of GCTB cells required a strict microenvironment and was highly selective in environmental factors. We hypothesized that GCTB with a high relapse rate and low rate of metastasis might be due to GCTB was more suitable to grow in the bone microenvironment.

It is necessary to search for factors related to tumor progression that may predict the clinical outcome of GCTB patients. Jaffe et al. classified GCTB as benign, aggressive and malignant based on the degree of histological appearance of the stromal cells and the number of giant cells and mitoses^29^. However, the histological staging system of Jaffe and its prognostic value of this grading were controversial. Previous reports showed that there were no correlation between prognosis and the histological grading of GCTB. Enneking and Campanacci et al. proposed a new classifications of GCTB based on clinical and radiographic features^6,7^. As our findings in the present study, some previous reports showed that there was no correlation between the risk of recurrence and the radiographic grading of GCTB, and the prognostic significance of the clinicoradiological staging systems described Enneking and Campanacci et al. were controversial^8–10^. One important aim of this study was to find factors that may be involved in the progression of the GCTBs and to identify potential targets for future diagnosis and therapy. We compared expression of ALCAM and patient outcome using a panel of 64 GCTB cases found that prognosis of GCTB patients was significantly associated with ALCAM expression.

In summary, we showed that ALCAM could be a novel prognostic expression signature for GCTB. The measurement of ALCAM expression should serve as a potential index of cancer-specific survival rates of patients with GCTB. More in-depth studies on the elucidation of the signal cascades and/or molecules modulating ALCAM should help to advance our understanding of the biology of GCTB. In addition, it is of special clinical interest that the risk index calculated from expression of ALCAM may be useful in future clinical practice.

## Materials and methods

### Flow cytometry

Cells were treated with a trypsin-EDTA solution. Cells were centrifuged at 1500 r.p.m. for 10 min and supernatants were discarded. Twenty microliter of PE-conjugated or FITC-conjugated antibodies(BD Pharmingen, San Diego, CA, USA) was then added to individual tubes and tubes were left in a 4 °C refrigerator in the dark for 30 min. Cells were washed twice with PBS. Cell pellets were re-suspended in 300 µl of PBS and analyzed with MACSQuant (Miltenyi Biotech Inc, Bergisch Gladbach, Germany). Isotype controls were included in all flow cytometry analyses. After cells were incubated for 30 min with the respective antibodies, propidium iodide staining was used to observe whether the number of dead cells increased. Flowjo v7.6.2 software (Treestar Inc, San Carlos, CA) was used for data analysis.

### Quantitative PCR

The sequences of PCR primers were listed in Table S2. Total RNA was extracted using TRIzol reagent (Invitrogen, Carlsbad, CA, USA) and reverse transcription was then conducted with PrimeScript RT reagent kit (Takara Bio Inc., Dalian, China).The quantitative PCR (qPCR) analyses were conducted to quantitate mRNA relative expression using SYBR Pre-mixExTaq (TaKaRa) with the 7900 real-time PCR system (Applied Biosystems). Beta-actin mRNA was used as an internal control.

### Cell culture and sphere formation

GCTB28 cells^30^ were cultured in DMEM containing 10% FBS. Cell culture medium was changed according to the conventional procedure. Cells were trypsinized and passaged once they grew to 70–80% confluence. Experimental cells were in the logarithmic growth phase. For the sphere formation assay, 10^5^ cells were placed on culture dishes coated with poly-HEMA (Sigma-Aldrich, St. Louis, MO). Spheres were cultured in DMEM/F12 serum-free medium (Invitrogen) supplemented with Epidermal Growth Factor (EGF, 20 ng/ml, PeproTech, USA), Fibroblast Growth Factor (FGF, 20 ng/ml, PeproTech,USA), Insulin-like Growth Factor (IGF, 20 ng/ml, PeproTech, USA). Adherent cells and spheres were incubated at 37˚C containing 5% CO_2_.

### Magnetic activated cell sorting (MACS)

An EDTA digestion solution (2.5 mmol/l EDTA and 1% FBS in PBS, pH 7.4) was used to treat cells at 4 °C for 30 min. Cells were then harvested and adjusted to a concentration of 1 × 10^7^ cells/ml. Cells were incubated at 4 °C for 10 min, rinsed in DF12 medium (Gibco, Bethesda,MD,USA), and pelleted by centrifugation. Anti-mouse IgG immunomagnetic beads were then added to the samples (Miltany Company, Bergisch Gladbach, Germany). Per instructions provided by the manufacturer’s instructions of Miltenyi Biotec MACS Cell Separation Kit (Miltenyi Biotech Inc, Bergisch Gladbach, Germany), cells were isolated by a MiniMACS separation column.

### Cell proliferation assay and colony formation

Cell Counting Kit-8 (CCK-8; Dojindo Laboratories, Japan) was used for cell proliferation. Cells (1 × 10^3^/well) were plated into 96-well plates. Ten microliters of CCK-8 solution were added to each well.The absorbance at 450 nm was detected after incubation at 37°C for 2 h. For the colony formation assays, cells (1 × 10^3^) were trypsinized and plated onto six- well plates and maintained in media containing 10% FBS for 10 days. Colonies were fixed with methanol and stained with 0.1% crystal violet in 20% methanol. The number of colonies was counted using an inverted microscope.

### Analysis of cell cycle

Cells in logarithmic growth were collected and fixed in 70% precooled ethanol at 4°C for 2 h. Following fixation, cells were centrifuged at 1000 r.p.m. for 10 min, washed with PBS twice, resuspended in 0.4 ml of PBS, and transferred into a new centrifuge tube. Then, cells were re-suspended in 500 ml of PBS containing 100 U/ml RNase A and incubated in a 37 °C water bath for 30 min. Fifty milliliter of propidium iodide (PI) were added into the suspension (5–50 mg/ml) followed by staining in the dark room (30 min, room temperature). The cell numbers for individual phases of the cell cycle were counted with flow cytometer (BD Bioscience, MA, USA).

### Cell migration and invasion assays

Cell migration and invasion assays were performed in a 24-well plate with 8 μm pore size transwell chamber (BD Biosciences, NJ, USA). For the migration assay, 5 × 10^4^ cells were seeded in the upper chamber of each transwell insert. For the invasion assay, the membrane was coated with Matrigel (BD Biosciences, Billerica, MA, USA) and 200 μl cell suspensions in culture medium containing 1 × 10^5^ cells was added to each 24-well invasion chamber. In each lower chamber, 800 μl of Dulbecco’s modified Eagle’s medium (DMEM) with 10% fetal bovine serum (FBS) was added as chemoattractant. After incubation overnight in a humidified tissue culture incubator at 37 °C, 5% CO_2_ atmosphere, the membrane inserts were removed from the plate, and non-invading cells in the upper surface were removed with a cotton swab moistened with medium. Cells that moved to the bottom surface of the chamber were stained with 0.1% crystal violet in 20% methanol. Cells were imaged and counted using an IX71 inverted microscope (Olympus, Tokyo, Japan).

### Apoptosis measurement

After cell collection, Annexin V apoptosis detection kit (Roche Diagnostics, Mannheim, Germany) was used to detect apoptosis by flow cytometry in accordance with the manufacturer’s instruction. Flowjo v7.6.2 software (Treestar Inc., San Carlos, CA)was used to analyze the test data.

### Lentiviral vector construction, packaging, and cell infection

The lentiviral shRNA vectors targeting ALCAM and scrambled control shRNA were purchased from Open Biosystems. HEK-293T cells were transfected with target plasmids along with the packaging and envelope plasmids psPAX2 and pMD2.G using Lipofectamine 2000 (Invitrogen) according to the manufacturer’s instructions. Virus particles were harvested 48 h after transfection. Cells were infected with recombinant lentivirus-transducing units plus 6 mg/ml polybrene (Sigma).

### Animal experiments

Four-to-five-week-old athymic nude mice (BALB/c-nu/nu) were anesthetized with pentobarbital sodium (20 mg/kg) by injection via gluteus maximus. GCTB28 cells suspension (DMEM and Matrigel (1:1), 20 µl) with gradient cell number was then slowly injected into the tibial marrow cavity via a micro-injector. Skyscan 1076 in vivo X-ray microtomograph (Skyscan Inc.,Belgium) and Kodak DXS 4000 Pro System (Carestream Health, Inc., CT, USA) X-ray scanning for the small animals were conducted to regularly observe the tumor growth in mice tibia. For in vivo pulmonary metastasis experiments, ALCAM^+^ and ALCAM^−^GCTB28 cells (luciferase-labeled) were intravenously injected respectively via tail vein of nude mice aged 6 weeks. Xenogen IVIS 100 cooled CCD camera (Xenogen, CA) was used to regularly detect the fluorescence in nude mice. During 2–12 weeks, these mice were anesthetized for fluorescence detection using in vivo imaging system. After these mice were sacrificed, the lungs were rapidly removed, fixed, paraffin-embedded, and sectioned for histopathological examination.

### Immunohistochemistry

Formalin-fixed, paraffin-embedded tissue samples were sectioned (4 lm thick) and mounted on charged slides. Immunohisto-chemical staining for ALCAM (DAKO, Carpinteria, CA), were performed using the streptavidin-biotin method. Negative controls, in which the primaryantibody was omitted, were also included in each run. Ten high-power fields ( × 400) of paraffin-embedded tissue section in each case included in the survival analysis were randomly selected to capture digital images. Image Pro Plus software (Media Cybernetics, USA) was used to analyze and determine the score based on the number and the staining intensity of the ALCAM^+^ cells.

### Source of clinical samples

In the present study, sampling and data collection of the clinical cases were from our institutes. Clinical information(Table S6) and tissue samples of 64 cases of GCTB from 2008 to 2011 were obtained, with >5 years of follow-up as of July 2016. Among the samples, 28 cases of clinical tumors and corresponding tumor-adjacent normal tissue were used to study the expression of ALCAM.

### Statistical analysis

SPSS17.0 software(SPSS Inc., Chicago, IL) was used for statistical analysis. Description of continuous variables was presented in mean ± s.d. Student’s *t*-test was used to compare the mRNA and protein expression in different samples. Chi-square (*χ*^2^) test and Fisher’s exact test were used to evaluate correlation between ALCAM expression of GCTB and clinicopathological features of the patients with GCTB. Survival analysis of the patients was conducted using Kaplan–Meier method and log rank test.

## Electronic supplementary material

Revised Supplemental data
