# Supplementary material for: ALCAM+ stromal cells: role in giant cell tumor of bone progression
Source: Cell Death Dis. 2018 Feb 20;9(3):299. doi: 10.1038/s41419-018-0361-z (PMC5833735; doi:10.1038/s41419-018-0361-z)
Supplement: Supplementary file 1 — Revised Supplemental data [file 41419_2018_361_MOESM1_ESM.doc]

**­Supplementary Information**

**Title**

ALCAM^+^ stromal cells: Role in giant cell tumor of bone progression.

**Authors**

Zhenhua Zhou^1†^, Yan Li^2†^, Xudong Wang^1†^, Jingjing Hu^3^, Muyu Kuang^4^, Zhiwei Wang^5^, Song Li^1^, Weidong Xu^5^*, Jianru Xiao^1^*

**Inventory of Supplemental Information**

Supplemental Figures(Figures S1-S5) and Figure legends

Supplemental Tables(Tables S1-S6) and Table legends

**Supplemental Figures**

**
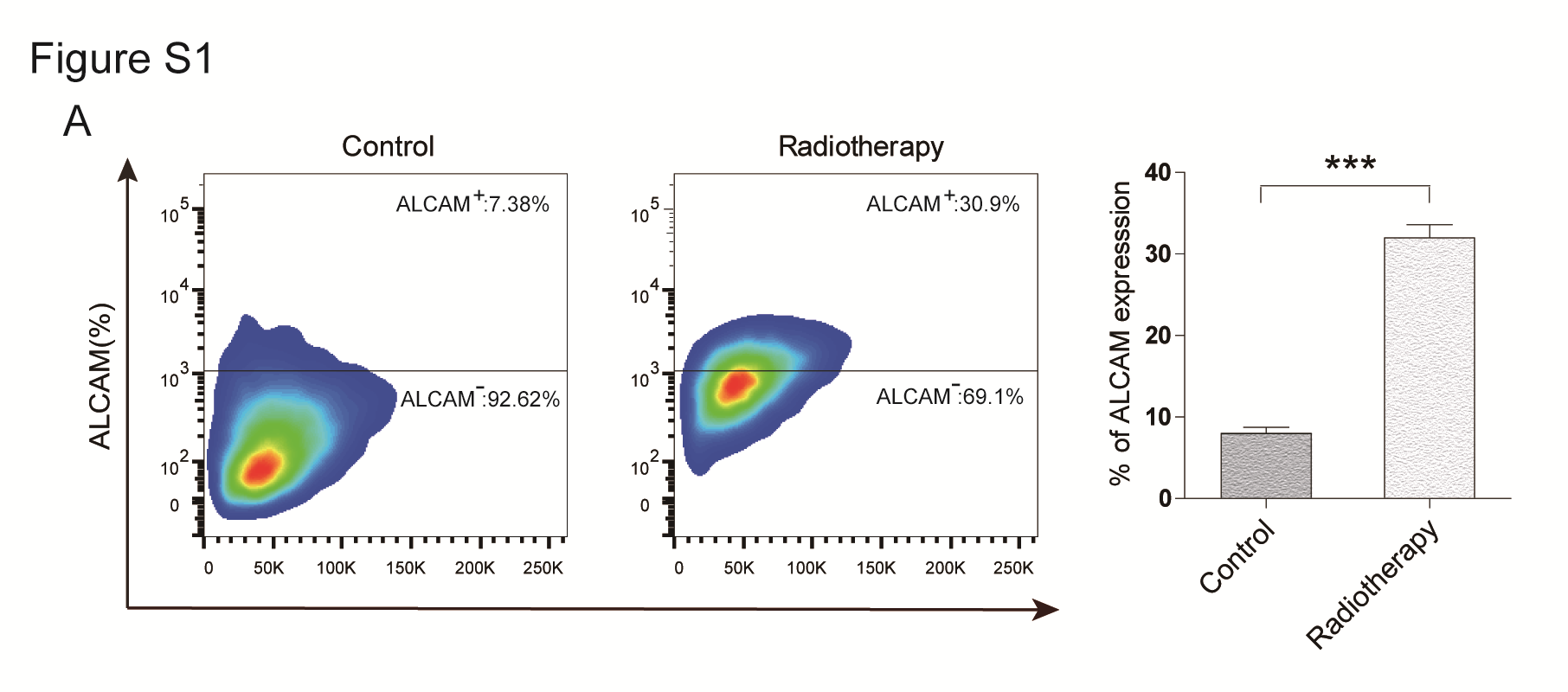
**

**Figure S1.Expression of ALCAM in GCTB cells after *in vitro* radiotherapy.**

Expressions of ALCAM in GCTB cells after radiotherapy were detected by flow cytometry; Independent experiments were repeated three times. Data were presented using mean ± standard deviation (t-test, ***: ***p*** < 0.001; **: ***p*** < 0.01; *: ***p*** < 0.05; NS: No Significance).

**
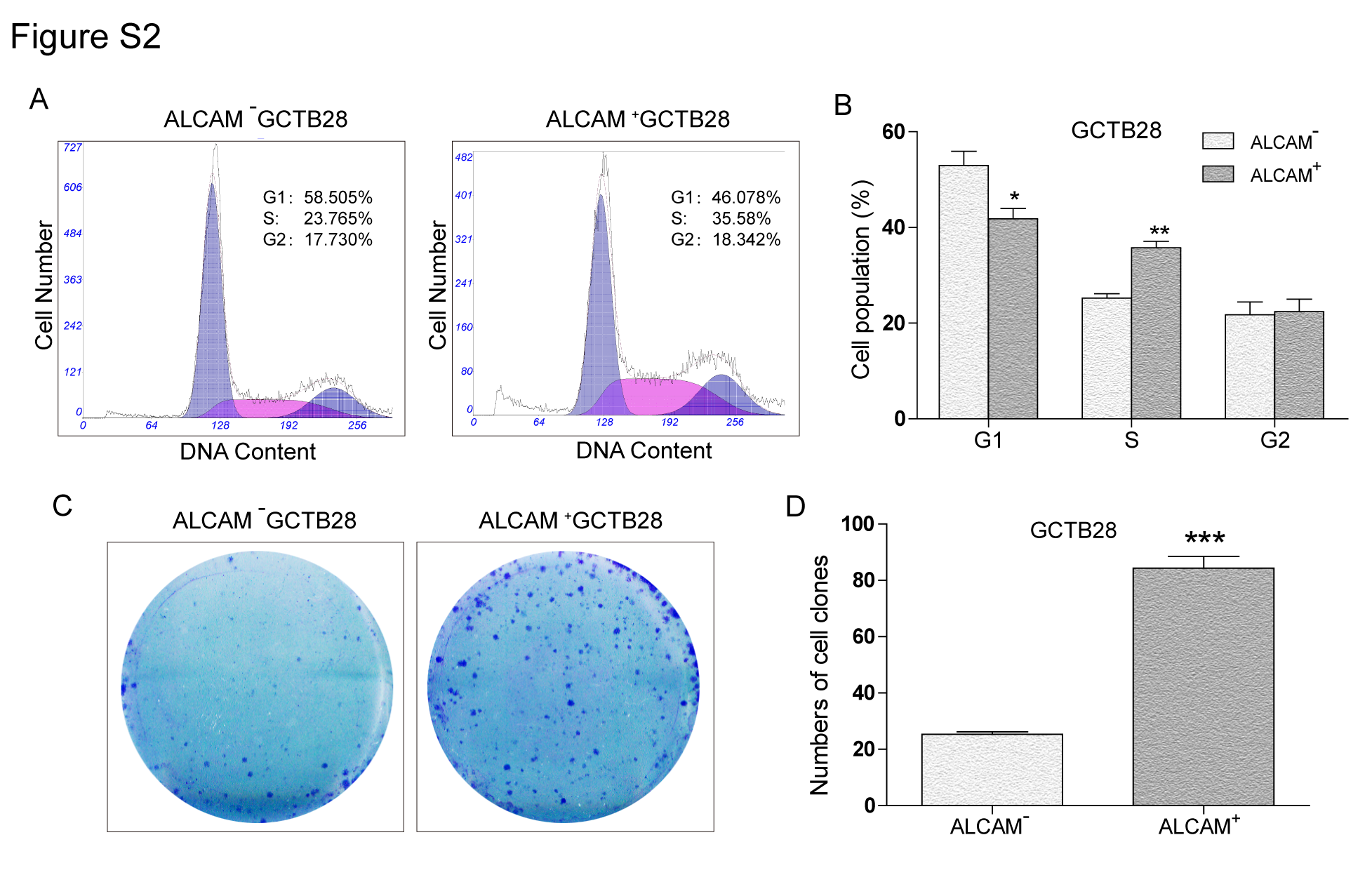
**

**Figure S2.** **ALCAM promotes GCTB cells proliferation *in vitro***

(A,B)The cell cycle of ALCAM^-^ and ALCAM^+^ cells of GCTB28 was analyzed by way of fluorescence-activated cell sorting (FACS). (C,D) Colony formation assays of ALCAM^-^ and ALCAM^+^ cells of GCTB28. The results are presented as the mean ± standard error of the mean of triplicate experiments. Statistical analysis was performed using the Student’s t-test. *P < 0.05; **P<0.01; ***P < 0.001.

**
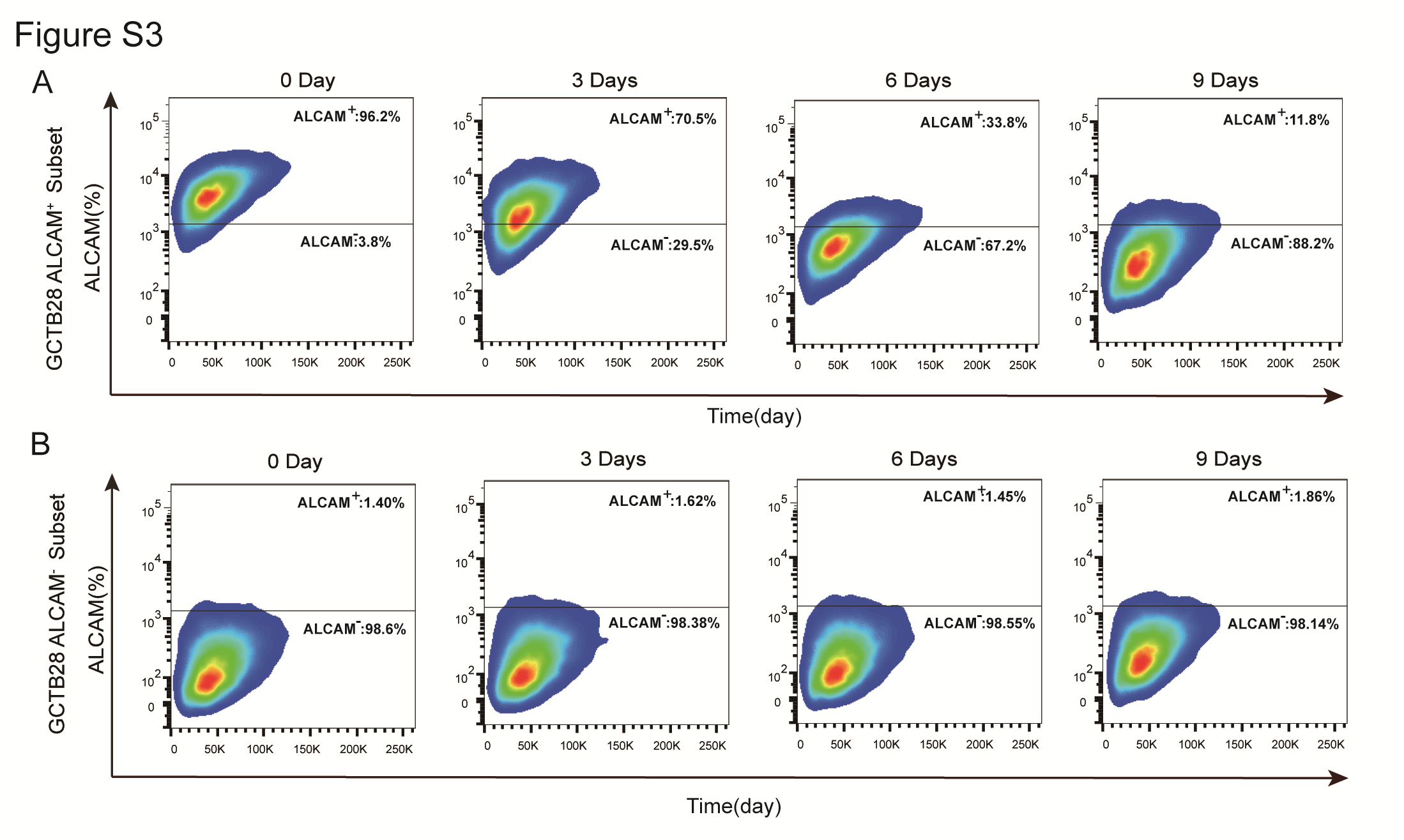
**

**Figure S3. ALCAM^+^ cells had the differentiate capacity to ALCAM^-^ cells**

**(A** and **B)** Variation of ALCAM expression in （**A**）ALCAM^+^ and （**B**）ALCAM^-^ subsets isolated from GCTB28 cells underwent conventional cell culture (10% FBS + normal adherent culture dish) were detected by flow cytometry at different time points (0, 3, 6, and 9 days); The results showed that the level of ALCAM expression in the ALCAM^+^ cells decreased from 96.2% to 11.8% in GCTB28 cells. However, the sorted ALCAM^-^ cells continued to maintained a stable low expression of ALCAM even two weeks later.


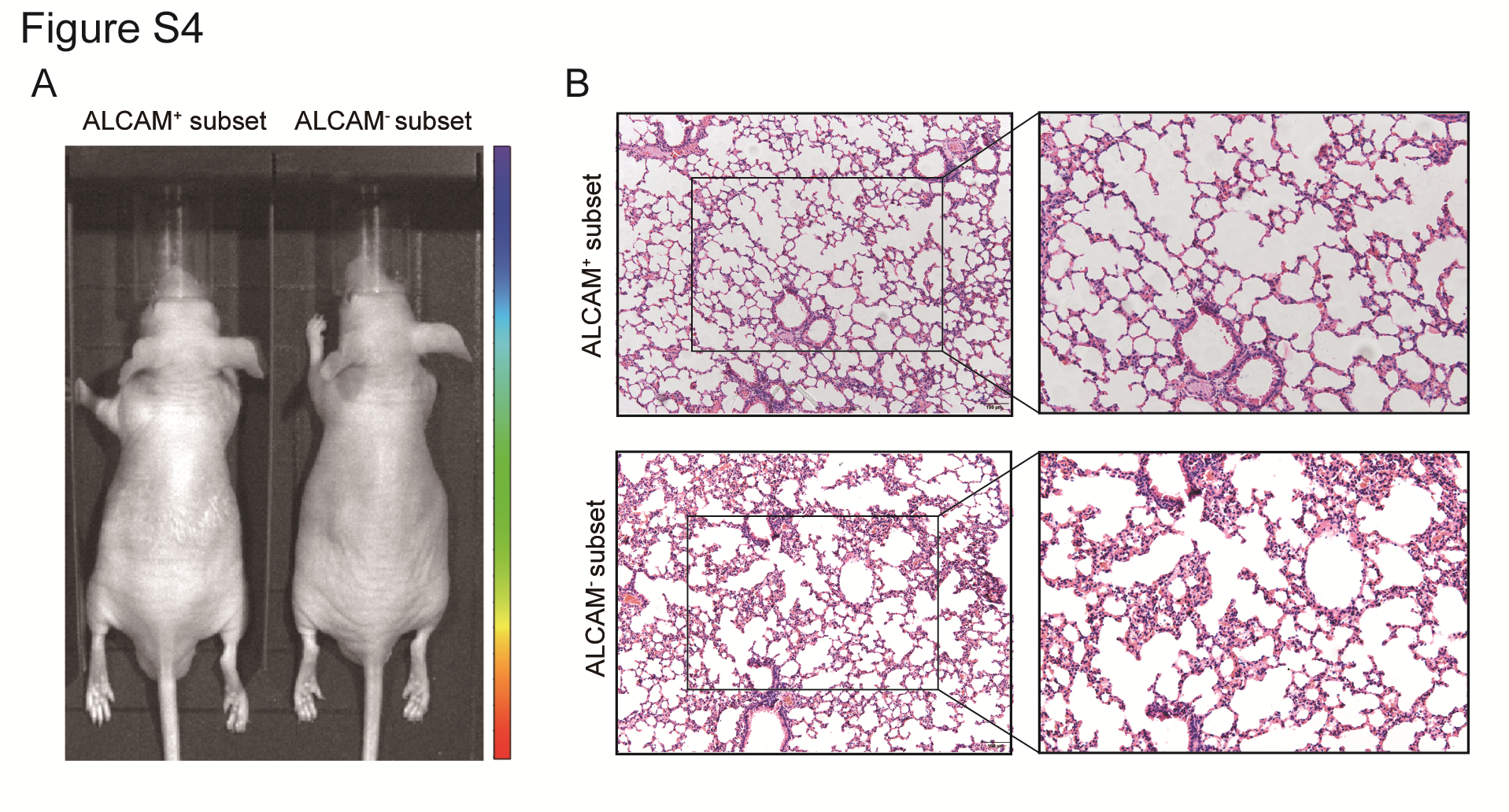
**Figure S4. Metastatic Capacity of ALCAM^+^ GCTB28 cells**

**(A** and **B)** By experimental metastatic assay in immunodeficient mice which was evaluated by bioluminescent imaging. Injecting intravenously ALCAM^+^ GCTB28 cells via tail vein of mice didn’t lead to development of metastasis *in vivo*. **(A)**Representative bioluminescent images of mice at 12 weeks after 1×10^6^ ALCAM^+^ and ALCAM^-^ cells injection respectively. **(B)** H&E staining of corresponding paraffin-embedded lung tissue from mice(Scale bar = 200 µm; Magnified: scale bar = 50 µm).

**
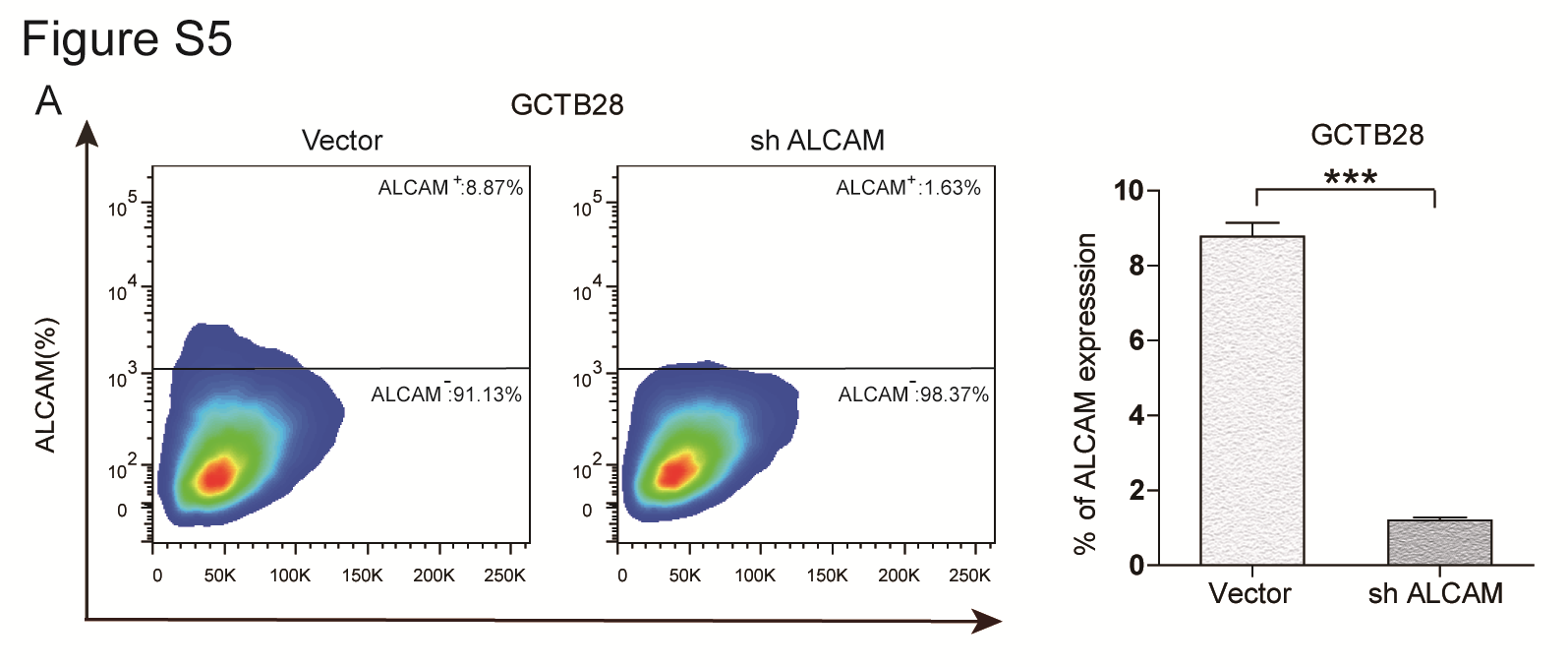
**

**Figure S5. Expresion of ALCAM in ALCAM knockdown cells.**

ALCAM knockdown experiments in GCTB28 cells was carried out by using lentiviral vectors.

Expresion of ALCAM in NTC and sh ALCAM cells was measured by using flow cytometry.

**Supplemental Tables**

**Table S1. Expression of various cell surface markers in different parental and corresponding sphere cells of GCTB28 cells**

|  | Parental | Sphere |  | Parental | Sphere |
| --- | --- | --- | --- | --- | --- |
| CD13 | 7.73±0.23 | 8.32±0.41 | CD45 | 1.26±0.14 | 2.33±0.21 |
| CD14 | 1.40±0.53 | 2.10±0.32 | CD59 | 51.2±0.98 | 60.2±0.77 |
| CD15 | 1.62±0.26 | 1.53±0.20 | CD71 | 1.86±0.22 | 2.96±0.34 |
| CD20 | 1.41±0.15 | 1.82±0.13 | CD73 | 48.8±1.01 | 52.3±1.28 |
| CD24 | 1.50±0.13 | 1.68±0.18 | CD90 | 96.9±1.98 | 99.1±2.96 |
| CD29 | 98.8±2.17 | 97.6±2.54 | CD106 | 1.82±0.20 | 1.76±0.23 |
| CD33 | 1.54±0.12 | 2.04±0.23 | CD117 | 2.87±0.27 | 2.53±0.25 |
| CD34 | 1.21±0.15 | 1.61±0.21 | CD133 | 2.18±0.31 | 2.69±0.35 |
| CD38 | 1.32±0.18 | 1.09±0.11 | CD166* | 7.56±0.77 | 80.28±2.45 |
| CD44 | 8.98±0.41 | 7.65±0.37 | CD221 | 1.03±0.11 | 1.70±0.16 |

Each surface marker expression was presented using percentage (%) as value unit and mean ± standard deviation (from three representative independent experiments).

*CD166 ( Also known as ALCAM).

**Table S2. Primers sequences for real-time PCR**

| **Name** | **Forward primer(5'-3')** | **Reverse primer(5'-3')** |
| --- | --- | --- |
| ***ALCAM*** | CTGGCAGTGGAAGCGTCATA | CGTCTGCCTCATCGTGTTCT |
| ***OCT-4*** | CTTGCTGCAGAAGTGGGTGGAGGAA | CTGCAGTGTGGGTTTCGGGCA |
| ***NANOG*** | AATACCTCAGCCTCCAGCAGATG | TGCGTCACACCATTGCTATTCTTC |
| ***SOX-2*** | AAATGGGAGGGGTGCAAAAGAGGAG | CAGCTGTCATTTGCTGTGGGTGATG |
| ***BMI-1*** | TGGAGAAGGAATGGTCCACTTC | GTGAGGAAACTGTGGATGAGGA |

**Table S3. Tumorigenicity of ALCAM^+^ and ALCAM^-^ GCTB cells**

| Cell line | Pheontypes | No.of Injected Cells | No. of Mice with Tumor Formation | | |
| --- | --- | --- | --- | --- | --- |
|  |  |  | 1 Months | 2 Months | 3 Months |
| GCTB28 | ALCAM^+^ | 500 | 0/5 | 0/5 | 0/5 |
|  |  | 1000 | 0/5 | 1/5 | 1/5 |
|  |  | 2500 | 2/5 | 3/5 | 4/5 |
|  |  | 10000 | 4/5 | 5/5 | - |
|  | ALCAM^-^ | 500 | 0/5 | 0/5 | 0/5 |
|  |  | 1000 | 0/5 | 0/5 | 0/5 |
|  |  | 2500 | 0/5 | 0/5 | 0/5 |
|  |  | 10000 | 0/5 | 0/5 | 1/5 |

**Table S4. Tumorigenicity of human GCTB cells after knocking out *ALCAM*** gene

| Cell line | Pheontypes | No.of Injected Cells | No.of Mice with Tumor Formation | | |
| --- | --- | --- | --- | --- | --- |
|  |  |  | 1 months | 2 months | 3months |
| GCTB28 | Nontarget | 1×10^3^ | 0/5 | 0/5 | 0/5 |
|  | Control cells | 5×10^3^ | 0/5 | 0/5 | 0/5 |
|  |  | 2.5×10^4^ | 1/5 | 2/5 | 2/5 |
|  |  | 1×10^5^ | 4/5 | 4/5 | 4/5 |
|  | sh ALCAM cells | 1×10^3^ | 0/5 | 0/5 | 0/5 |
|  |  | 5×10^3^ | 0/5 | 0/5 | 0/5 |
|  |  | 2.5×10^4^ | 0/5 | 0/5 | 0/5 |
|  |  | 1×10^5^ | 0/5 | 0/5 | 0/5 |

**Table S5. Correlation of ALCAM expression with clinico-pathologic features in GCTB patients**

| ***Features*** | ***Number of cases*** | ***Level of ALCAM*** | | ***χ²*** | ***P*** |
| --- | --- | --- | --- | --- | --- |
|  |  | **Low(n,%)** | **high(n,%)** |  |  |
| **Age** |  |  |  |  |  |
| **Age≤30** | 30 | 24(80.0%) | 6(20.0%) | 0.753^a^ | 0.386^a^ |
| **Age＞30** | 34 | 24(70.6%) | 10(29.4%) |  |  |
| **Gender** |  |  |  |  |  |
| **Male** | 28 | 20(71.4%) | 8(28.6%) | 0.339^a^ | 0.561^a^ |
| **Female** | 36 | 28(77.8%) | 8(22.2%) |  |  |
| **Enneking stages** |  |  |  |  |  |
| **II** | 14 | 9(64.3%) | 5(35.7%) | 1.097^b^ | 0.295^b^ |
| **III** | 50 | 39(78.0%) | 11(22.0%) |  |  |
| **Jaffe stages** |  |  |  |  |  |
| **I/II** | 54 | 40(74.1%) | 14(25.9%) | 0.158^b^ | 0.691^b^ |
| **III** | 10 | 8(80.0%) | 2(20.0%) |  |  |
| **Tumor relapse** |  |  |  |  |  |
| **Absence** | 44 | 37(84.1%) | 7(15.9%) | 6.206^a^ | 0.013^a^ |
| **Presence** | 20 | 11(55.0%) | 9(45.0%) |  |  |

a：*χ²* test; b：Fisher’s exact test.

**Table S6. Clinical Data of a Series of 64 Cases**

| **No.** | **Gender** | **Age（yr）** | **Enneking stages** | **Jaffe stages** | **Level of ALCAM** | **Relapse** |
| --- | --- | --- | --- | --- | --- | --- |
| 1 | Male | 46 | III | I/II | Low | Absence |
| 2 | Female | 38 | III | I/II | High | Absence |
| 3 | Female | 44 | II | I/II | High | Presence |
| 4 | Male | 21 | III | I/II | Low | Absence |
| 5 | Male | 20 | III | III | Low | Presence |
| 6 | Male | 18 | III | I/II | Low | Absence |
| 7 | Male | 21 | III | I/II | Low | Absence |
| 8 | Male | 40 | III | III | High | Absence |
| 9 | Female | 25 | III | III | Low | Absence |
| 10 | Male | 50 | III | III | Low | Absence |
| 11 | Female | 52 | III | I/II | Low | Presence |
| 12 | Male | 61 | II | I/II | Low | Absence |
| 13 | Male | 34 | III | III | Low | Presence |
| 14 | Male | 35 | III | I/II | Low | Absence |
| 15 | Female | 26 | III | I/II | Low | Presence |
| 16 | Male | 32 | III | III | Low | Presence |
| 17 | Female | 40 | III | I/II | Low | Absence |
| 18 | Male | 43 | II | I/II | High | Presence |
| 19 | Male | 38 | III | I/II | Low | Absence |
| 20 | Female | 22 | III | I/II | High | Absence |
| 21 | Male | 19 | III | I/II | High | Presence |
| 22 | Male | 27 | III | I/II | Low | Absence |
| 23 | Male | 23 | III | I/II | Low | Absence |
| 24 | Female | 19 | III | I/II | Low | Presence |
| 25 | Female | 32 | II | I/II | Low | Absence |
| 26 | Male | 37 | II | I/II | Low | Absence |
| 27 | Male | 35 | III | I/II | High | Absence |
| 28 | Male | 42 | III | I/II | Low | Presence |
| 29 | Male | 24 | II | I/II | High | Presence |
| 30 | Female | 51 | III | I/II | Low | Absence |
| 31 | Male | 49 | II | I/II | Low | Absence |
| 32 | Male | 25 | III | III | Low | Presence |
| 33 | Male | 29 | III | I/II | Low | Absence |
| 34 | Female | 32 | II | I/II | High | Presence |
| 35 | Male | 32 | III | I/II | Low | Absence |
| 36 | Female | 45 | II | I/II | Low | Absence |
| 37 | Male | 42 | III | I/II | Low | Presence |
| 38 | Female | 47 | III | I/II | High | Absence |
| 39 | Male | 26 | III | I/II | Low | Absence |
| 40 | Female | 28 | III | I/II | Low | Absence |
| 41 | Female | 21 | III | I/II | Low | Presence |
| 42 | Male | 20 | II | I/II | Low | Absence |
| 43 | Female | 33 | III | I/II | Low | Absence |
| 44 | Male | 24 | III | III | Low | Absence |
| 45 | Male | 21 | III | I/II | Low | Absence |
| 46 | Female | 36 | III | I/II | High | Presence |
| 47 | Male | 27 | III | I/II | High | Presence |
| 48 | Female | 25 | III | I/II | Low | Absence |
| 49 | Male | 22 | II | I/II | Low | Presence |
| 50 | Female | 22 | III | I/II | Low | Absence |
| 51 | Female | 27 | III | I/II | Low | Absence |
| 52 | Female | 38 | III | III | High | Presence |
| 53 | Male | 37 | III | I/II | Low | Absence |
| 54 | Female | 61 | II | I/II | Low | Absence |
| 55 | Male | 25 | III | I/II | High | Absence |
| 56 | Female | 55 | III | I/II | Low | Absence |
| 57 | Female | 57 | II | I/II | High | Presence |
| 58 | Male | 42 | III | I/II | Low | Absence |
| 59 | Male | 18 | III | I/II | High | Absence |
| 60 | Female | 19 | III | I/II | Low | Absence |
| 61 | Female | 36 | III | I/II | Low | Absence |
| 62 | Male | 24 | III | I/II | Low | Absence |
| 63 | Female | 44 | II | III | Low | Absence |
| 64 | Female | 21 | III | I/II | Low | Absence |
